# Age and fixation strategy as associated factors for sacroiliac joint dysfunction after posterior pelvic ring fixation

**DOI:** 10.3389/fsurg.2025.1719425

**Published:** 2025-12-15

**Authors:** Cemil Aktan, Çile Aktan, Muhammed Ergün, Halil Yalçın Yüksel, Alpaslan Erol

**Affiliations:** 1 Department of Orthopaedics and Traumatology, Antalya Training and Research Hospital, Antalya, Türkiye; 2 Department of Algology Clinic, Antalya Training and Research Hospital, Antalya, Türkiye

**Keywords:** sacroiliac joint dysfunction, pelvic ring injury, sacroiliac screw fixation, unilateral fixation, bilateral fixation, functional outcomes

## Abstract

**Background:**

Sacroiliac joint dysfunction (SIJD) is an underrecognized source of postoperative pain and disability after pelvic ring stabilization. Although percutaneous sacroiliac screw fixation provides stable fixation with low morbidity, it restricts physiological SI joint micromotion, potentially causing iatrogenic dysfunction. The relative contributions of injury severity vs. fixation strategy to SIJD remain poorly defined.

**Objectives:**

To investigate the incidence and factors associated with SIJD after sacroiliac screw fixation for posterior pelvic ring injuries, focusing on fixation laterality (unilateral vs. bilateral) and patient characteristics. Secondary aims included evaluating the functional impact of SIJD on long-term outcomes.

**Methods:**

This single-center retrospective cohort study with prospective follow-up included 80 patients [mean follow-up: 42.3 ± 27.3 months; median 36 months (IQR: 16–56)] who underwent sacroiliac screw fixation between 2016 and 2024. Fracture morphology was classified using Tile and Young–Burgess systems. SIJD was diagnosed prospectively based on ≥3 positive provocation tests. Functional outcomes were assessed using the Oswestry Disability Index (ODI), Visual Analog Scale (VAS), and Lower Extremity Functional Scale (LEFS). Statistical analyses included chi-square, Mann–Whitney *U*, Cohen's *κ*, effect sizes (Cohen's *d*), and multivariate logistic regression.

**Results:**

SIJD occurred in 20 patients (25%), exclusively after unilateral fixation, whereas none followed bilateral fixation (*p* = 0.004). SIJD-positive patients were younger (32.1 ± 9.0 vs. 41.5 ± 12.8 years, *p* < 0.001). Fracture morphology showed no consistent association. At ≥12-month follow-up, SIJD-positive patients had worse outcomes (ODI: 37.3 vs. 10.3; VAS: 5.7 vs. 1.6; LEFS: 33.5 vs. 70.3; all *p* < 0.001). Multivariate analysis identified younger age as an independent predictor (OR 1.8 per decade, *p* = 0.026).

**Conclusions:**

Unilateral fixation is associated with a higher incidence of SIJD, particularly in younger patients. Given the retrospective design, findings should be interpreted as associative and hypothesis-generating rather than confirmatory. Fixation strategy, rather than fracture morphology, appears to be the key associated factor. SIJD causes substantial long-term disability and pain, underscoring the need for individualized fixation planning and prospective validation.

**Level of evidence:**

Level III (Retrospective cohort with prospective follow-up).

## Introduction

The sacroiliac joint (SIJ) is a complex synovial–ligamentous structure that transmits axial loads from the spine to the pelvis while allowing minimal physiological micromotion (<2 mm). Its unique composition—hyaline cartilage on the sacral side, fibrocartilage on the iliac side, and strong ligamentous support—ensures lumbopelvic stability but also predisposes the joint to dysfunction under abnormal mechanical stress (1–4). Sacroiliac joint dysfunction (SIJD) is an underrecognized source of persistent pain and disability, with etiologies including trauma, degenerative changes, and iatrogenic factors (2, 5).

Posterior pelvic ring injuries, often resulting from high-energy trauma, are frequently stabilized using percutaneous sacroiliac screw fixation due to its minimally invasive nature and biomechanical effectiveness (6–8). However, this technique directly crosses the SIJ, altering its biomechanics by rigidly restricting its natural micromotion. Recent studies suggest that such alterations may contribute to iatrogenic SIJD and chronic pain during long-term follow-up (9–11). Distinguishing dysfunction attributable to the initial injury from that associated with fixation remains a critical but underexplored issue in pelvic trauma surgery.

Existing literature has identified several potential factors associated with post-fixation SIJD, including implant positioning, number of screws, and bilateral fixation, but evidence remains limited and heterogeneous. Age-related changes in pelvic morphology and joint compliance may further influence outcomes, yet this factor has received little attention. Moreover, most studies have focused primarily on radiographic parameters, often without standardized functional assessments (7, 8).

Therefore, this study aimed to investigate the association between sacroiliac screw fixation and the occurrence of SIJD following posterior pelvic ring injuries. Specifically, we examined the roles of age, sex, and fixation strategy (unilateral vs. bilateral) in the incidence of SIJD. Functional outcomes were compared between patients who did and did not develop SIJD using the Oswestry Disability Index (ODI), Visual Analog Scale (VAS), and Lower Extremity Functional Scale (LEFS) scores. By clarifying the associations between fixation-related factors and SIJD, this study seeks to highlight the importance of patient selection and fixation planning, and to inform potential strategies such as alternative implant trajectories, selective screw removal, or SIJ fusion in selected cases.

## Materials and methods

### Study design and participants

This was a single-center retrospective cohort study with a prospective observational follow-up. All patients who underwent SIJ screw fixation for posterior pelvic ring fractures at the Orthopaedics and Traumatology Department of Antalya Training and Research Hospital between 2016 and 2024 were identified from hospital records. Patients were contacted and invited to outpatient visits, where standardized SIJD provocation tests and functional assessments were conducted. Those who attended the follow-up and signed informed consent were included in the prospective phase.

Fracture pattern and fixation characteristics were reviewed retrospectively from operative notes and radiographs to enable analysis of both injury-related and fixation-related factors associated with SIJD.

The study protocol was approved by the institutional ethics committee (Approval No: 9/8, Date: 29 May 2025) and all procedures were conducted in accordance with the Helsinki Declaration.

### Eligibility criteria

**Figure 1:**
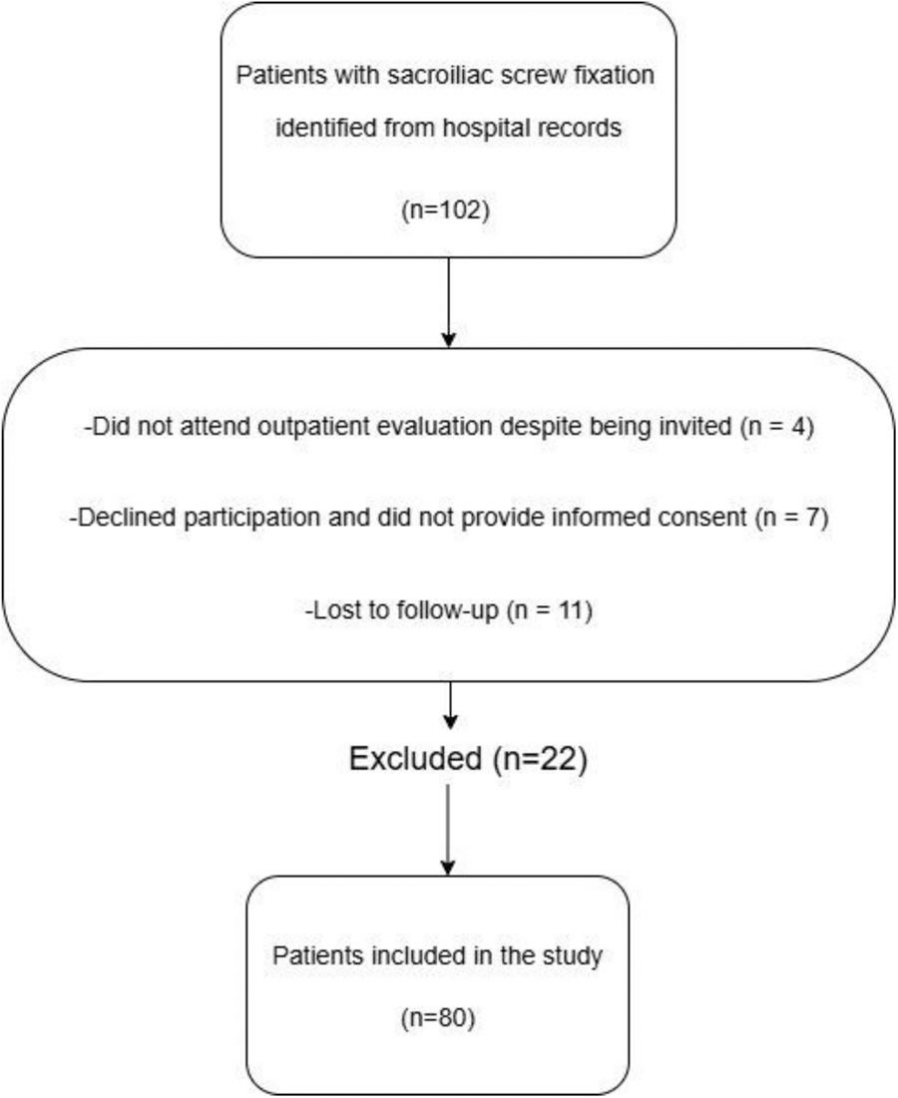
Flow diagram of patient selection and inclusion process. Flow diagram of patient selection. Of 102 patients identified with sacroiliac screw fixation, 22 were excluded due to non-attendance at follow-up (*n* = 4), refusal to participate (*n* = 7), or loss to follow-up (*n* = 11). The final study cohort comprised 80 patients.

A total of 102 patients who had previously undergone unilateral or bilateral sacroiliac screw fixation for posterior pelvic ring fractures were screened for eligibility. Of these, 80 patients fulfilled the inclusion criteria and completed both the retrospective review and the prospective follow-up assessments. Eligible participants were adults aged ≥18 years, who had undergone sacroiliac screw fixation with a minimum postoperative follow-up of 12 months and who attended the outpatient evaluation to complete standardized SIJD provocation tests. Exclusion criteria were refusal to participate, incomplete demographic or clinical records, or a follow-up duration shorter than 12 months (Figure 1). Patients who did not provide complete outcome data during follow-up were excluded from the final statistical analysis; therefore, a complete-case analysis approach was adopted, and no data imputation was performed.

### Surgical technic

All sacroiliac screw fixations were performed percutaneously under fluoroscopic guidance by the same pelvic trauma surgical team. Patients were positioned supine on a radiolucent operating table, and closed reduction of the posterior pelvic ring was achieved prior to fixation. Standard 7.3 mm cannulated screws with a 32 mm thread length (overall length 75–105 mm) were inserted through the ilium across the sacroiliac joint into the sacral body, with screw length and trajectory individualized according to fracture morphology and patient anatomy. The entry point and screw placement were confirmed using anteroposterior, inlet, and outlet fluoroscopic views.

Intraoperative fluoroscopy routinely included anteroposterior, lateral, inlet, and outlet projections to ensure proper screw trajectory and avoid foraminal penetration. Postoperative imaging consisted of standardized AP, inlet, and outlet pelvic radiographs for all patients, which provided adequate visualization of screw trajectory and foraminal integrity. CT was reserved for cases in which fluoroscopic or radiographic findings suggested possible malposition (*n* = 14), and no foraminal or extraosseous penetration was identified. Representative intraoperative fluoroscopic and postoperative radiographic images are presented in Supplementary Figure S1.

**Figure 2:**
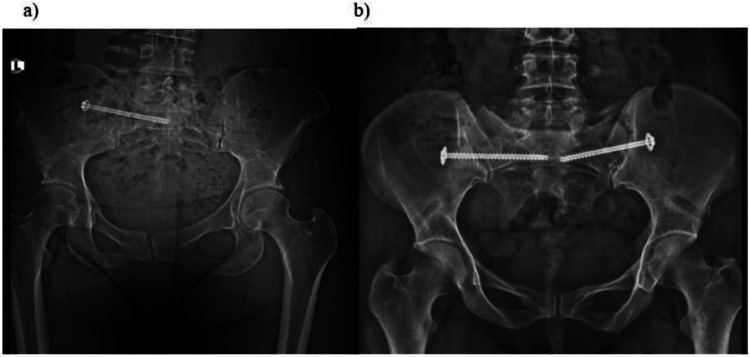
Anteroposterior pelvic radiographs showing sacroiliac screw fixation configurations. **(a)** Unilateral sacroiliac screw fixation. **(b)** Bilateral sacroiliac screw fixation.

Unilateral or bilateral fixation was selected based on the stability requirements of the injury pattern. In bilateral fixation cases, standard 7.3-mm cannulated iliosacral screws were placed at the S1 level on both right and left sides. No long trans-sacral screws, S2-only screws, combined S1–S2 constructs, or triangular/HA-coated implants were used in this cohort. All procedures were performed without open reduction, bone grafting, or additional posterior instrumentation (Figure 2).

All fractures were classified independently by two senior orthopedic surgeons from the same surgical team, without knowledge of each other's assessments, according to the Tile (9) and Young–Burgess (10) systems. Inter-observer reliability was assessed using Cohen's kappa (*κ*) value with a 95% confidence interval calculated separately for patients with and without SIJD. Any discrepancies were resolved by consensus prior to statistical analysis.

In addition to fracture classification, fixation characteristics were reviewed retrospectively from operative reports and intraoperative fluoroscopic images. These included the number of screws (single vs. double), level of fixation (S1 only vs. combined S1–S2), and laterality (unilateral vs. bilateral). Screw positioning was assessed based on intraoperative fluoroscopic inlet, outlet, and lateral projections; however, postoperative evaluation relied primarily on anteroposterior pelvic radiographs.

### Provocation tests for sacroiliac dysfunction

All diagnostic provocation tests were performed by the same experienced pain specialist who was blinded to the fixation strategy, fracture classification, and other clinical and radiological data, to ensure inter-observer consistency. Sacroiliac joint dysfunction (SIJD) was diagnosed when at least three of the following tests elicited pain: Gaenslen test, Thigh Thrust test, Fortin's finger test, Distraction test, Compression test, and FABER test. Each test was performed bilaterally, except for Fortin's finger test, which was conducted on the side previously identified as painful during the other provocation maneuvers. This diagnostic criterion is supported by previous studies demonstrating that a cluster of ≥3 positive provocation tests yields high diagnostic accuracy, with reported sensitivity of approximately 94% (11, 12). All tests were performed after the completion of radiographic and clinical data collection to minimize diagnostic bias. In patients with bilateral screw fixation, tests were interpreted separately for each side based on pain localization during maneuvers.

### Clinical assessments

Functional outcomes were evaluated using three validated instruments. Disability in daily activities was assessed with the Oswestry Disability Index (0–100), with higher scores indicating greater disability (13). Pain intensity was measured using the Visual Analog Scale (0–10), where 0 represents no pain and 10 represents the worst imaginable pain (14). Lower extremity function was assessed with the Lower Extremity Functional Scale (0–80), in which higher scores reflect better functional capacity (15).

All functional assessments were performed at the final outpatient follow-up visit, at least 12 months postoperatively, to minimize variability due to short-term recovery. Evaluations were performed by an independent orthopedic surgeon who was blinded to fracture classification, fixation strategy, and radiological findings.

Patients were stratified into SIJD-positive and SIJD-negative groups based on the provocation test criteria, and comparative analyses of ODI, VAS, and LEFS scores were conducted to examine the functional associations of SIJD. In cases with bilateral fixation, functional scores were assigned according to the side that demonstrated positive provocation tests and predominant symptoms.

Baseline functional scores prior to injury were not available; therefore, comparisons focused on postoperative differences between SIJD-positive and SIJD-negative groups.

### Statistical analysis

Patients with incomplete outcome data or who did not complete the follow-up evaluation were excluded from the final analysis; therefore, statistical analyses were performed using a complete-case approach without data imputation. All statistical analyses were performed using IBM SPSS Statistics for Windows, Version 26.0 (IBM Corp., Armonk, NY, USA). The normality of continuous variables was assessed using the Shapiro–Wilk test and visual inspection of histograms. Continuous variables were expressed as mean ± standard deviation (SD) and compared between groups using the Student's *t*-test or Mann–Whitney *U* test, as appropriate. For variables with non-normal distributions, median and interquartile range (IQR) were additionally reported. Follow-up duration demonstrated right-skew (mean > median); therefore, both mean ± SD and median (IQR) are presented. Categorical variables were expressed as counts and percentages, and differences between groups were evaluated using the chi-square test or Fisher's exact test when expected frequencies were <5.

Interobserver reliability for fracture classification systems was assessed using Cohen's kappa (*κ*) coefficient with corresponding 95% confidence intervals (CI) and interpreted according to Landis and Koch's benchmarks (*κ*: 0.61–0.80 = substantial; *κ* > 0.80 = excellent). Effect sizes for functional outcome measures (ODI, VAS, and LEFS) were calculated using Cohen's *d* and interpreted according to conventional benchmarks (small = 0.2, medium = 0.5, large = 0.8).

Multivariate logistic regression analysis was performed to examine factors associated with SIJD, including age, sex, fracture classification, and fixation laterality. Odds ratios (OR) and 95% confidence intervals (CI) were calculated. Because complete separation was observed for fixation laterality (no SIJD events in the bilateral group), Firth's penalized logistic regression was additionally fitted to obtain bias-reduced, finite estimates. Results from both the penalized and conventional models are reported in parallel. Model performance was summarized using the c-statistic for discrimination and the Hosmer–Lemeshow test for calibration, and multicollinearity was screened with variance inflation factors (VIF <5).

A *post-hoc* power analysis was also conducted (two-proportion framework; G*Power 3.1 equivalent) based on the observed SIJD proportions for unilateral (20/62) vs. bilateral (0/18) fixation. The achieved power (1 − *β*) was approximately 0.99 at *α* = 0.05 (two-tailed), indicating adequate sensitivity to detect the between-group difference. Detailed parameters of this analysis are presented in Supplementary Table S1.

All tests were two-tailed, and a *p* value <0.05 was considered statistically significant.

## Results

A total of 80 patients were included in the analysis, comprising 60 SIJD-negative and 20 SIJD-positive individuals. Demographic characteristics, including age, sex distribution, BMI, and follow-up duration, are summarized in Table 1. Patients with SIJD were significantly younger than those without SIJD (*p* < 0.001). Median follow-up duration for the whole cohort was 36 months (IQR: 16–56 months).

**Table 1:** Demographic and fixation Side comparison of patients With and without SIJD.

Characteristic	SIJD (-) (*n* = 60)	SIJD (+) (*n* = 20)	*p*-value
Sex (M/F)	34/26	7/13	*p* = 0.16
Age (Mean ± SD)	41.50 ± 12.80	32.10 ± 9.00	*p* < 0.001
Postoperative Duration (Mean ± SD) (months)	41.80 ± 28.60	43.70 ± 26.40	*p* = 0.79
Fixation Side—Right (R)	26	10	—
Fixation Side—Left (L)	16	10	—
Fixation Side—(R, L)	18	0	—
BMI (kg/m^2^)	25.10 ± 2.50	26.40 ± 3.80	*p* = 0.17

Demographic characteristics and fixation side distribution of patients with and without sacroiliac joint dysfunction (SIJD). Data are presented as mean ± standard deviation (SD) or number of patients (%), unless otherwise specified. *P*-values were calculated using Student's *t*-test or chi-square test, as appropriate.

Comorbidity profiles were similar in both groups. The prevalence of hypertension, diabetes mellitus, and cardiac disorders showed no significant differences (all *p* ≥ 0.57). COPD was slightly more common among SIJD-negative patients, without statistical significance (*p* = 0.57), and no cases of asthma were observed in either group. The younger age profile of SIJD-positive patients indicates that fixation-related factors, rather than degenerative changes, were more frequently associated with dysfunction.

Postoperative imaging consisted primarily of standard AP pelvic radiographs, which were routinely available in patient records. Additional CT scans had been obtained in 14 patients (17.5%) at the time of their original clinical management when screw malposition was suspected. Review of these scans revealed no cases of foraminal or extraosseous screw penetration.

Provocation test results showed clear and clinically relevant differences between groups. In the SIJD-negative cohort, 22 patients (36.7%) had no positive tests, 25 (41.7%) had a single positive test, and 13 (21.7%) exhibited two positive tests; none met the diagnostic threshold of ≥3 tests. In contrast, all SIJD-positive patients demonstrated multiple test positivity: 2 patients (10.0%) were positive for four tests, 14 (70.0%) for five tests, and 4 (20.0%) for all six tests.

Notably, in every SIJD-positive case, all positive provocation tests were ipsilateral to the side of screw placement, with no contralateral dysfunction observed (100% concordance, *p* < 0.001, binomial test). The analysis was based on the side of fixation, as the study did not classify patients according to the side or severity of the initial sacroiliac injury. Therefore, no definitive conclusions can be drawn regarding the relationship between SIJD laterality and the side of injury.

**Figure 3:**
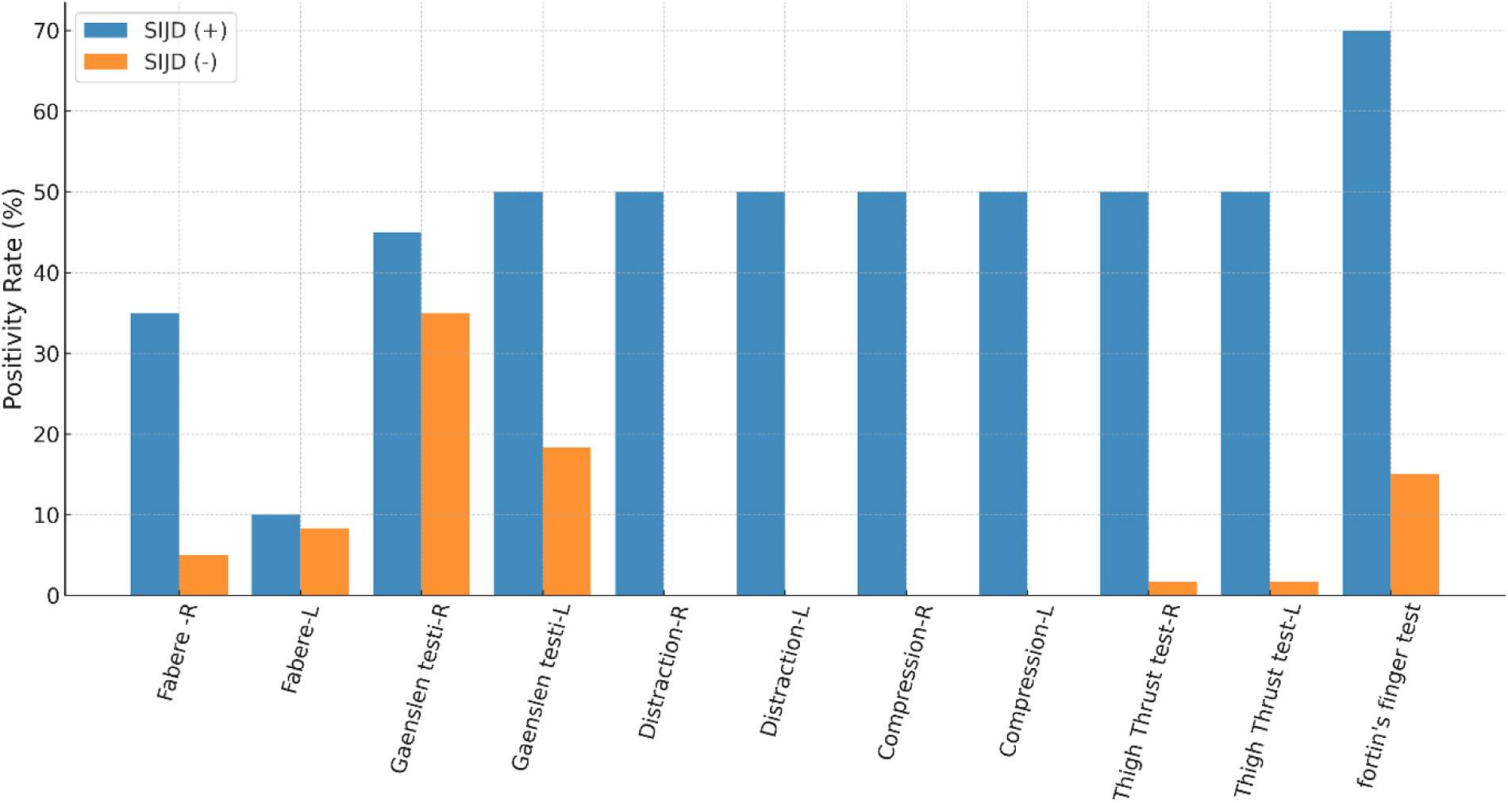
Comparison of provocation test positivity between SIJD-positive and SIJD-negative patients. Distraction, compression, and thigh thrust tests were universally positive in SIJD-positive patients and nearly absent in the SIJD-negative group. Fortin's finger test also showed substantially higher positivity in the SIJD-positive group (70% vs. 15%). In contrast, false-positive responses in the SIJD-negative group were observed most frequently with the Gaenslen test (35%), and to a lesser extent with Fortin's finger (15%) and FABER (8.3%).

Among individual maneuvers, distraction, compression, and thigh thrust tests were universally positive in SIJD-positive patients, highlighting their diagnostic strength, while Fortin's finger test showed high discriminative value (70%) (Figure 3). In contrast, false-positive responses in the SIJD-negative group were most frequent with the Gaenslen test (35%), followed by Fortin's finger (15%) and FABER (8.3%), whereas distraction, compression, and thigh thrust were rarely positive in non-SIJD patients. These findings confirm the high diagnostic accuracy of clustered provocation testing and underscore the strong laterality between screw placement and dysfunction.

Fracture types were classified according to the Tile and Young–Burgess systems. Interobserver agreement for both systems was consistently high in patients with and without SIJD. For the Tile system, *κ* was 0.86 (95% CI: 0.67–1.00) in SIJD-negative patients and 0.83 (95% CI: 0.60–1.00) in SIJD-positive patients. For the Young–Burgess system, *κ* values were 0.85 (95% CI: 0.73–0.97) and 0.86 (95% CI: 0.67–1.00), respectively. The overlapping confidence intervals indicated excellent reproducibility, with no significant differences in classification reliability between SIJD-positive and SIJD-negative groups (Table 2).

**Table 2:** Interobserver reliability for fracture classification systems in patients with and without SIJD.

Classification system	Group	*κ* (Cohen's kappa)	95% CI
Tile	SIJD (−)	0.86	0.67–1.00
	SIJD (+)	0.83	0.60–1.00
Young–Burgess	SIJD (−)	0.85	0.73–0.97
	SIJD (+)	0.86	0.67–1.00

Interobserver reliability for Tile and Young–Burgess fracture classification systems in patients with and without sacroiliac joint dysfunction (SIJD). Values are presented as Cohen's kappa (*κ*) with 95% confidence intervals (CI).

95% confidence intervals for Cohen's kappa were calculated using the asymptotic standard error. Upper limits exceeding 1.00 were truncated to 1.00 by convention.

After resolving classification discrepancies by consensus, fracture subtype distributions were analyzed. According to the Tile system, type B fractures predominated in both SIJD-negative and SIJD-positive groups, particularly B2 (45.0% vs. 55.0%), followed by B1 (8.3% vs. 20.0%) and B3 (20.0% vs. 0%). Type C fractures were less common overall, comprising C1 (10.0% vs. 20.0%), C2 (6.7% vs. 5.0%), and C3 (10.0% vs. 0%). Among these, only B3 fractures showed a statistically significant difference, occurring exclusively in the SIJD-negative group (*p* = 0.031).

Based on the Young–Burgess system, lateral compression (LC) injuries were the most frequent pattern, dominated by LC2 (46.7% vs. 45.0%), followed by LC3 (33.3% vs. 0%) and LC1 (6.7% vs. 30.0%). Anterior–posterior compression (APC) injuries included AP2 (10.0% vs. 10.0%) and AP3 (3.3% vs. 10.0%), whereas vertical shear (VS) injuries were rare (0% vs. 5.0%).

Statistically significant intergroup differences were observed for LC1, which was more frequent in SIJD-positive patients (*p* = 0.013), and LC3, which occurred only in SIJD-negative patients (*p* = 0.002). No other subtypes showed significant differences (all *p* > 0.05) (Table 3).

**Table 3:** Distribution of Tile and Young–Burgess fracture subtypes between SIJD− and SIJD+ groups.

Classification	Subtype	SIJD− (*n* = 60)	SIJD+ (*n* = 20)	*p*-value
Tile	B1	5 (8.3%)	4 (20.0%)	0.217
	B2	27 (45.0%)	11 (55.0%)	0.605
	B3	12 (20.0%)	0 (0.0%)	0.031
	C1	6 (10.0%)	4 (20.0%)	0.258
	C2	4 (6.7%)	1 (5.0%)	1.000
	C3	6 (10.0%)	0 (0.0%)	0.328
Young–Burgess	AP2	6 (10.0%)	2 (10.0%)	1.000
	AP3	2 (3.3%)	2 (10.0%)	0.259
	LC1	4 (6.7%)	6 (30.0%)	0.013
	LC2	28 (46.7%)	9 (45.0%)	1.000
	LC3	20 (33.3%)	0 (0.0%)	0.002
	VS	0 (0.0%)	1 (5.0%)	0.250

Distribution of Tile and Young–Burgess fracture subtypes in patients with and without sacroiliac joint dysfunction (SIJD). Data are presented as *n* (%). *P*-values were calculated using Fisher's exact test or chi-square test, as appropriate. Statistically significant values are shown in bold.

Fixation laterality showed a significant relationship with SIJD development. All SIJD cases occurred following unilateral fixation, whereas none were observed after bilateral fixation (SIJD+: 0/20 vs. SIJD−: 18/60; *p* = 0.004, Fisher's exact test). A *post-hoc* power analysis based on these proportions (unilateral: 20/62 vs. bilateral: 0/18) indicated high achieved power (1 − *β* ≈ 0.99, *α* = 0.05, two-tailed) for detecting the between-group difference in SIJD incidence (Supplementary Table S1). This finding indicates that SIJD was more frequently associated with unilateral fixation, whereas bilateral fixation appeared less commonly linked to dysfunction, although causality cannot be inferred due to the retrospective design.

Multivariate logistic regression analysis, including age, sex, fracture classification, and fixation laterality, identified age as an independent predictor of SIJD (per decade increase, OR: 1.8, 95% CI: 1.1–3.0, *p* = 0.026). Because complete separation precluded inclusion of fixation laterality, an additional Firth's penalized logistic regression confirmed consistent directionality of the age effect (penalized OR = 1.7, 95% CI: 1.0–2.9). Due to the retrospective nature of the study and the limited number of SIJD events, the multivariable model was restricted to a small number of clinically relevant covariates (age, sex, fracture classification, and fixation laterality) to avoid overfitting.

At a minimum follow-up of 12 months, patients who developed SIJD exhibited markedly worse functional outcomes compared with those without SIJD. ODI scores were significantly higher in the SIJD-positive group (37.3 ± 10.3 vs. 10.3 ± 4.6), as were VAS scores (5.7 ± 1.2 vs. 1.6 ± 1.0), indicating greater disability and pain levels. Conversely, LEFS scores were substantially lower in SIJD-positive patients (33.5 ± 5.9 vs. 70.3 ± 11.7), reflecting significantly impaired lower extremity function. All group differences were highly significant (*p* < 0.001, Mann–Whitney *U* test).

Effect size analyses demonstrated very large effects for all outcome measures (ODI: *d* = 4.08; VAS: *d* = 3.90; LEFS: *d* = 3.62) (Table 4). The mean between-group differences in ODI and LEFS also exceeded the established minimal clinically important difference (MCID) thresholds, confirming that the detected differences were clinically substantial.

**Table 4:** Comparison of functional (ODI, LEFS) and pain (VAS) scores between SIJD-positive and SIJD-negative patients at ≥12-month follow-up.

Measure	SIJD− (*n* = 60)	SIJD+ (*n* = 20)	*p*-value	Cohen's *d*
ODI	10.30 ± 4.60	37.30 ± 10.30	<0.001	4.08
VAS	1.60 ± 1.00	5.70 ± 1.20	<0.001	3.90
LEFS	70.30 ± 11.70	33.50 ± 5.90	<0.001	3.62

Values are presented as mean ± standard deviation (SD). *P*-values were calculated using the Mann–Whitney *U* test. Effect sizes were expressed as Cohen's *d*, with all measures showing very large differences between groups.

### Complications

A total of four patients experienced perioperative complications, accounting for 5% of cases. Two patients (2.5%) developed Trendelenburg gait due to superior gluteal nerve injury confirmed during follow-up, and two patients (2.5%) experienced intraoperative bleeding caused by injury to a branch of the superior gluteal artery, which was promptly controlled with cauterization without the need for further surgical intervention. In the postoperative period, four patients (5%) developed wound drainage, and three patients (3.75%) exhibited implant loosening at the final follow-up. Notably, all cases of implant loosening occurred in the SIJD-negative group. No cases of implant or wound infection were detected during the follow-up period.

## Discussion

In this retrospective cohort with prospective follow-up, sacroiliac joint dysfunction was observed in one quarter of patients after sacroiliac screw fixation for posterior pelvic ring injuries. SIJD occurred exclusively after unilateral fixation and was consistently ipsilateral to the fixation side. Younger age was significantly associated with SIJD, whereas the higher frequency among women did not reach statistical significance. These findings indicate that both fixation strategy (unilateral vs. bilateral S1 screw fixation) and patient-specific factors such as age are strongly associated with postoperative SIJD.

Provocation maneuvers proved to be highly effective diagnostic tools. Distraction, compression, and thigh thrust tests were consistently positive among SIJD-positive patients, whereas Gaenslen's test produced the highest rate of false positives, confirming its limited specificity. These results align with previous studies and emphasize that clustered maneuvers are substantially more reliable than single tests for diagnosing SIJD (4, 11).

Posterior pelvic ring injuries remain among the most challenging conditions in orthopedic trauma, and percutaneous sacroiliac screw fixation is widely regarded as the gold standard for achieving stable fixation with low morbidity (16–18). Although minimally invasive, this technique alters joint biomechanics by crossing the SI joint, which has been associated with postoperative dysfunction. In our cohort, SIJD was observed only after unilateral fixation, whereas no cases were detected after bilateral fixation. This pattern is generally consistent with biomechanical findings indicating that unilateral iliosacral screw fixation provides comparatively lower construct stiffness and may lead to increased sacroiliac joint motion under load compared with more rigid multi-screw or trans-sacral configurations (19–21). However, we agree with the reviewer that the stability provided by a single short, partially threaded S1 iliosacral screw is inherently lower than that achieved with long trans-sacral, multi-screw, or triangular constructs. This reduced stiffness—combined with a physiologically mobile contralateral SI joint—may partially explain the relatively high SIJD incidence (25%) in our cohort. Importantly, none of the SIJD-positive patients demonstrated screw loosening or hardware failure, suggesting that dysfunction resulted not from mechanical failure but from altered joint mechanics and constrained micromotion at the fixed joint. Therefore, the observed association between unilateral fixation and SIJD should be interpreted within the specific biomechanical context of short unilateral S1 screws, and cannot be generalized to more rigid fixation configurations.

Patient factors also appear influential. Previous studies have suggested that female sex, lower BMI, and hormonal influences may predispose to SIJ pathology (22, 23), particularly in peripartum women, but in our cohort these factors were not statistically decisive. Younger patients were at significantly higher risk, which may reflect greater physiological SIJ mobility and higher mechanical demands. The younger age profile of SIJD-positive patients suggests that fixation-related biomechanical factors, rather than age-related degenerative changes, may play a more dominant role in the development of postoperative SIJD. Taken together, these findings suggest that fixation strategy and patient age are more closely associated with postoperative SIJ function than sex or BMI.

Fracture morphology did not show a consistent association with SIJD. Type B and LC patterns predominated in both groups, but specific subtypes such as B3 and LC1 did not demonstrate a clear or systematic relationship with dysfunction. This suggests that biomechanical alterations introduced by fixation, rather than fracture configuration itself, are the primary drivers of postoperative SIJ dysfunction.

It is also important to acknowledge that sacroiliac joint dysfunction may develop as a sequela of the initial pelvic injury itself, independent of fixation. However, in our cohort, patients with bilateral sacroiliac injuries, typically indicative of more severe trauma, were treated with bilateral fixation, and none of these patients developed SIJD during follow-up.

Although our results demonstrated that SIJD developed exclusively on the side of fixation, it remains biomechanically plausible that unilateral rigidity may increase stress transmission to the contralateral SI joint. However, no contralateral SIJD was identified in our series, suggesting that local joint mechanics and direct articular constraint, rather than contralateral overload, may play the predominant role.

This observation has important biomechanical implications. Because the patients requiring bilateral fixation also sustained bilateral posterior pelvic ring injuries, one would expect a higher inherent risk of SIJ irritation attributable to the severity of trauma alone. Counterintuitively, the complete absence of SIJD in this subgroup, contrasted with the exclusive occurrence of SIJD following unilateral fixation, suggests that the asymmetric load distribution and restriction of physiological micromotion introduced by unilateral constructs may play a more dominant role in the development of postoperative SIJ dysfunction than the injury pattern itself.

Although this interpretation remains associative due to the retrospective design, the consistent ipsilateral laterality pattern reinforces the hypothesis that fixation-related biomechanical asymmetry is a key contributing factor.

This finding supports an association between fixation strategy and postoperative SIJD, although causality cannot be inferred due to methodological limitations.

SIJD was associated with markedly poorer long-term functional outcomes. At a minimum follow-up of 12 months, SIJD-positive patients exhibited significantly higher ODI and VAS scores and lower LEFS scores, with very large effect sizes. These differences were both statistically and clinically meaningful, underscoring SIJD as an underrecognized but relevant postoperative sequela with substantial quality-of-life implications. The observed functional differences were not only statistically significant but also clinically meaningful. The mean between-group difference in ODI (≈27 points) clearly exceeded the reported minimal clinically important difference (MCID) threshold of 13–15 points for this scale, while the difference in LEFS (≈37 points) similarly surpassed the established MCID of 9 points. These findings suggest that the functional impairment associated with postoperative SIJD is substantial and of clear clinical relevance, rather than representing a merely statistical distinction (24).

Management strategies for SIJD vary. While conservative measures such as physical therapy or SIJ injections are first-line, surgical fusion is often required for refractory cases, typically using screw-based fixation or triangular titanium implants (25, 26). In our series, unilateral fixation was associated with a 25% incidence of SIJD, whereas no cases occurred after bilateral fixation, consistent with the reported biomechanical advantage of bilateral S1 iliosacral screw constructs, which provide greater rigidity and rotational stability than unilateral fixation. Waters et al. similarly reported higher rates of contralateral fusion after unilateral procedures (27). Interestingly, all SIJD cases in our cohort were ipsilateral to the fixation side, suggesting that local joint mechanics rather than contralateral overload play the dominant role (28). Importantly, no contralateral SIJD was identified in any patient, further reinforcing a consistent and reproducible laterality pattern.

The 25% incidence rate observed in our series is higher than that reported in most previous studies, which have generally shown rates of approximately 10% (29). Several factors may account for this discrepancy. Our study employed a standardized cluster of provocation maneuvers and validated functional outcome measures, likely improving detection sensitivity compared with earlier reports relying mainly on subjective symptoms. Moreover, the minimum follow-up period of 12 months may have captured late-onset SIJ dysfunction, and the younger age profile of our cohort could have further contributed to the higher observed rate.

Our cohort did not include isolated S2 fixation or combined S1–S2 constructs; therefore, direct comparison with biomechanical studies evaluating multilevel or long trans-sacral fixation was not feasible. The biomechanical implications of these alternative constructs remain important but fall outside the scope of the present dataset.

Screw loosening was observed in 5% of patients, all in the SIJD-negative group. This finding indicates that loosening may be related to mechanical fatigue and osseous fusion and may not be directly associated with SIJD. Notably, the absence of SIJD among patients with screw loosening may indicate that once solid fusion occurs, the risk of iatrogenic dysfunction decreases. However, this interpretation should be made cautiously given the retrospective design and low event numbers. Emerging strategies such as elective screw removal, non-transarticular fixation, or implants designed to promote fusion may help reduce late dysfunction, but prospective studies are needed to clarify their role (30).

This study has several important strengths. It represents, to our knowledge, the first retrospective study with prospective clinical follow-up evaluating SIJD after sacroiliac screw fixation. The direct comparison of unilateral and bilateral fixation strategies, the use of validated functional outcome instruments (VAS, ODI, LEFS), and systematic fracture classification using both Tile and Young–Burgess systems provide robust methodological support. These elements enhance both the internal validity and the clinical applicability of our findings.

Several limitations should be acknowledged. Its single-centre design and modest sample size may limit generalisability. The absence of advanced dynamic MRI prevented detailed biomechanical evaluation of sacroiliac joint motion and long-term changes. The minimum follow-up of 12 months may not fully capture late degenerative processes. The absence of preoperative baseline SIJ function limits our ability to distinguish injury-related from fixation-related dysfunction. Furthermore, pre-injury functional scores (ODI, VAS, and LEFS) were not available due to the retrospective design, which precludes assessment of absolute recovery. In addition, although standard 7.3-mm cannulated S1 screws (overall length 75–105 mm) were used in all cases, the absence of longer trans-sacral may represent a biomechanical limitation, particularly in patients with complex injury patterns or reduced bone quality. However, no screw breakage, migration, or fixation failure attributable to screw length was observed clinically or radiographically in our cohort. Nevertheless, as all patients were evaluated at standardized postoperative intervals, the observed between-group differences reliably reflect the relative functional impact of SIJD.

Despite these limitations, the study provides prospective evidence linking sacroiliac fixation strategy with functional outcomes and identifying key risk factors for iatrogenic SIJD.

### Clinical perspective

In patients with bilateral sacroiliac injuries, stable fixation across both joints can be achieved more easily, potentially reducing postoperative dysfunction. However, in unilateral injuries, the risk of sacroiliac joint dysfunction should be carefully considered when planning fixation. In such cases, alternative techniques that avoid transarticular screw placement—such as bridge plating across the posterior ring—or elective screw removal after solid union may be considered to better preserve joint biomechanics. These strategies warrant further investigation through prospective biomechanical and clinical studies to clarify their potential role in minimizing iatrogenic SI joint dysfunction.

Future studies should include larger, multicentre cohorts with longer follow-up to better define fixation-related risk factors and incorporate advanced biomechanical imaging or motion analyses for comparison of unilateral and bilateral constructs. Research into the long-term effects of screw removal and alternative fixation strategies may further inform evidence-based guidelines aimed at minimizing SIJD and optimizing functional outcomes.

## Conclusion

This study identifies an association between unilateral sacroiliac screw fixation and a higher incidence of sacroiliac joint dysfunction compared with bilateral fixation. These findings highlight the importance of individualized surgical planning that accounts for patient age, pelvic morphology, and fixation configuration in order to optimize long-term functional outcomes.

Surgical strategies should aim not only to restore anatomical stability but also to preserve physiological joint biomechanics, thereby minimizing the risk of iatrogenic SIJD.

While bilateral or trans-sacral fixation may be advantageous in patients with bilateral injuries or high biomechanical demands, alternative approaches—such as bridge plating or elective screw removal after union—could be considered in selected unilateral cases to reduce postoperative dysfunction.

Future prospective and biomechanical studies are needed to refine fixation techniques and to establish evidence-based guidelines for reducing postoperative dysfunction.

Given the retrospective design, the results should be interpreted as associative and hypothesis-generating rather than confirmatory.

## Data Availability

The raw data supporting the conclusions of this article will be made available by the authors, without undue reservation.
