# Supplementary material for: Age and fixation strategy as associated factors for sacroiliac joint dysfunction after posterior pelvic ring fixation
Source: Front Surg. 2025 Dec 15;12:1719425. doi: 10.3389/fsurg.2025.1719425 (PMC12745429; doi:10.3389/fsurg.2025.1719425)
Supplement: Supplementary file 1 [file Table1.docx]

**Supplementary Table S1**. Post-hoc power analysis parameters for SIJD incidence comparison (unilateral vs bilateral fixation)

| Parameter | Value |
| --- | --- |
| Test type | Two-proportion difference (two-tailed) |
| Significance level (α) | 0.05 |
| Group sizes | Unilateral: **n = 62**; Bilateral: **n = 18** |
| SIJD events | Unilateral: **20**; Bilateral: **0** |
| Observed proportions (p₁, p₂) | **0.323**, **0.000** |
| Effect size (Cohen’s h) | **1.21** (calculated from arcsin √p transformation) |
| Achieved power (1–β) | **≈ 0.99** |
| Power calculation method | Two-proportion test, G*Power 3.1 equivalent |
| Interpretation | The achieved power of ≈0.99 indicates sufficient sensitivity to detect the observed difference between groups; however, given the retrospective design, the analysis is interpreted as **supporting context** rather than confirmatory evidence. |
