# Supplementary figures and images for: Age and fixation strategy as associated factors for sacroiliac joint dysfunction after posterior pelvic ring fixation
Source: Front Surg. 2025 Dec 15;12:1719425. doi: 10.3389/fsurg.2025.1719425 (PMC12745429; doi:10.3389/fsurg.2025.1719425)

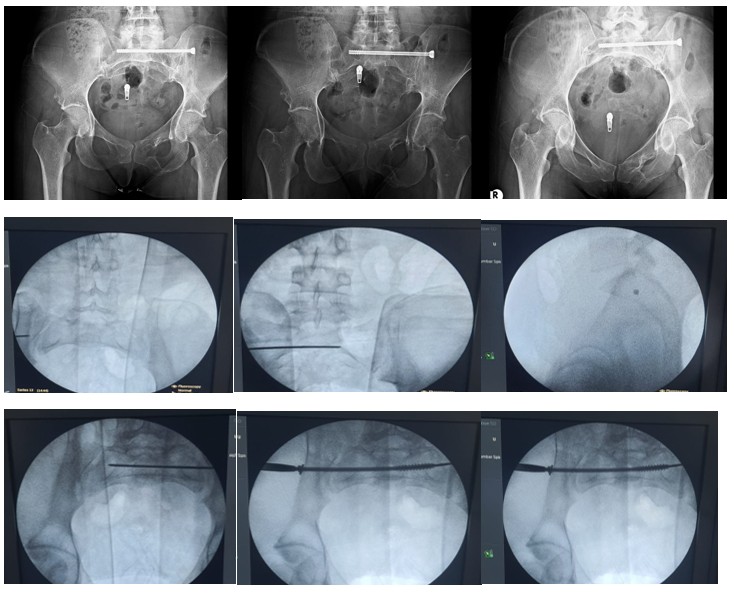

Supplement: Supplementary file 2 [file Image1.jpeg]
